# Level of FACT defines the transcriptional landscape and aggressive phenotype of breast cancer cells

**DOI:** 10.18632/oncotarget.15656

**Published:** 2017-02-23

**Authors:** Daria Fleyshman, Laura Prendergast, Alfiya Safina, Geraldine Paszkiewicz, Mairead Commane, Kelsey Morgan, Kristopher Attwood, Katerina Gurova

**Affiliations:** ^1^ Department of Cell Stress Biology, Roswell Park Cancer Institute, Buffalo, NY, USA; ^2^ Department of Biostatistics, University of Buffalo, SUNY, Buffalo, NY, USA

**Keywords:** FACT, SSRP1, SPT16, curaxin CBL0137, breast cancer

## Abstract

Although breast cancer (BrCa) may be detected at an early stage, there is a shortage of markers that predict tumor aggressiveness and a lack of targeted therapies. Histone chaperone FACT, expressed in a limited number of normal cells, is overexpressed in different types of cancer, including BrCa. Recently, we found that FACT expression in BrCa correlates with markers of aggressive BrCa, which prompted us to explore the consequences of FACT inhibition in BrCa cells with varying levels of FACT.

FACT inhibition using a small molecule or shRNA caused reduced growth and viability of all BrCa cells tested. Phenotypic changes were more severe in high- FACT cells (death or growth arrest) than in low-FACT cells (decreased proliferation). Though inhibition had no effect on the rate of general transcription, expression of individual genes was changed in a cell-specific manner. Initially distinct transcriptional profiles of BrCa cells became similar upon equalizing FACT expression. In high-FACT cells, FACT supports expression of genes involved in the regulation of cell cycle, DNA replication, maintenance of an undifferentiated cell state and regulated by the activity of several proto-oncogenes. In low-FACT cells, the presence of FACT reduces expression of genes encoding enzymes of steroid metabolism that are characteristic of differentiated mammary epithelia.

Thus, we propose that FACT is both a marker and a target of aggressive BrCa cells, whose inhibition results in the death of BrCa or convertion of them to a less aggressive subtype.

## INTRODUCTION

Advancements in the screening and diagnosis of breast cancer (BrCa) have led to the increased detection of tumors at a pre-invasive or early invasive stage, commonly leading to clinical intervention before the disease has a chance to spread. There are several subtypes of BrCa defined by the presence of molecular markers, such as Her2 and hormone receptors, which are used to predict the aggressive potential of BrCa and dictate therapeutic intervention. Though these markers have revolutionized the way clinicians approach BrCa, there is still significant heterogeneity within subtypes, which leads to the over-treatment or under-treatment of many patients whose tumors do not progress as projected by their molecular subtype.

Identification of novel predictive markers of aggressive BrCa within subtypes would have a major clinical advantage. We have found that levels of FAcilitates Chromatin Transcription (FACT) complex in BrCa correlate with poor overall survival, presence of clinical markers of bad prognosis (e.g. high grade of disease, triple negative status, HER2 amplification, and absence of estrogen receptor), and high probability of metastatic disease [1, 2]. Knockdown of FACT using RNAi inhibits tumor transformation and compromises the viability of tumor cells, but is well tolerated by non-tumor cells [1].

FACT, a complex of two subunits, SSRP1 and SPT16, belongs to a class of nuclear factors, known as histone chaperones that, as suggested by their name, serve one class of proteins - histones. Histones are the most basic, positively charged proteins known, which makes their attraction to DNA very high, but to each other quite low. Histones not only bind DNA, but form a highly organized complex of 8 subunits, known as a nucleosome core, which DNA wraps 1.65 times [3]. This highly organized process is possible due to histone chaperones [4]. They escort histones from the moment of their synthesis in the cytoplasm through several steps of oligomerization and post-translational modifications to the stage of presenting them to DNA for final nucleosome assembly. Different members of the histone chaperone family serve at different steps of this complex process and are involved in chromatin assembly, disassembly and maintenance [4]. Not surprisingly, histone chaperones were traditionally viewed as ubiquitously expressed housekeeping factors. However, more and more published studies describe differential expression of histone chaperones in normal and cancer tissues and the particular importance of some family members for the growth of tumor, but not normal cells [5–9]. In contrast, some histone chaperones are lost in several types of cancer, suggesting their roles as tumor suppressor genes [10, 11]. Deciphering which aspects of histone chaperone activity have pro- and anti-cancer activity is critically important for understanding their function in normal and diseased conditions as well as to explore some of them as targets for cancer treatment.

FACT is involved in almost all chromatin related processes, including DNA replication [12], repair [13] and transcription [14–16]. However, the most well characterized FACT function is to assist RNA polymerase (RNAP) elongation through chromatin [17]. Additionally, a deficit of FACT in yeast caused cryptic transcription, suggesting a role for FACT in stabilizing nucleosomes during transcription. [18] Structural studies suggest that FACT forms dynamic contacts with the histone core that weakens the core contact with DNA, thus facilitating RNA polymerase passage. At the same time, FACT protects the core from falling apart [19], [20]. Thus, the most probable function of FACT *in vivo* is to increase the efficiency of transcription while preserving chromatin structure.

Several years ago, we found that the anticancer activity of a class of small molecules known as curaxins is dependent on the functional inactivation of the FACT complex [21]. Further characterization of FACT triggered by this discovery led to the observation that FACT is not ubiquitously expressed in mammals. Moreover, it was detectable at the protein level in a very limited number of adult cells [22]. FACT is highly expressed at early stages of embryonic development with gradual reduction towards birth and postnatal expression in organs, such as bone marrow, immune and reproductive organs, bottom of intestinal crypts, suggesting the role of FACT in the maintenance of the undifferentiated cell state. This was confirmed by *in vitro* induced differentiation experiments [22]. Several studies from other labs also showed that FACT is involved in the early steps of differentiation [23, 24], suggesting a role in actively proliferating progenitors of differentiated cells, the most probable source of cancer stem cells. Based on these findings, FACT elevation in multiple tumors, including BrCa [25] and ovarian cancer [26] was less surprising and more biologically explicable, though the mechanism by which FACT facilitates tumor growth is still obscure.

FACT is not a DNA - binding transcription factor or a member of any known pathways. Its role was demonstrated mostly in chromatin related processes, such as transcription, general [14, 17, 19, 27] or gene specific [28], replication [12, 29], DNA repair [30], [13] and even mitosis [31] in different model systems. At the same time, none of these processes are universally dependent on FACT, because some normal cells do not express FACT and inhibition of FACT expression in normal cells that do express FACT does not significantly interfere with their viability and growth [1, 22]. To identify the mechanism(s) that explains tumor cell dependence on FACT among these plethora of possibilities, we aimed first to understand what phenotypical traits are associated with FACT expression in cancer cells. To achieve this, we used a panel of BrCa cell lines with varying FACT levels and analyzed the differences between “high” and “low”-FACT expressing cells. In addition, we assessed the sensitivity of these cell lines to FACT knockdown. Since the lead curaxin CBL0137, the first indirect FACT inhibitor, is currently in clinical trials, we addressed the important question concerning the consequences of FACT inactivation in “high” and “low” FACT expressing tumor cells.

## RESULTS

### Inhibition of FACT is lethal for BrCa cells with high basal FACT expression

**Figure 1:**
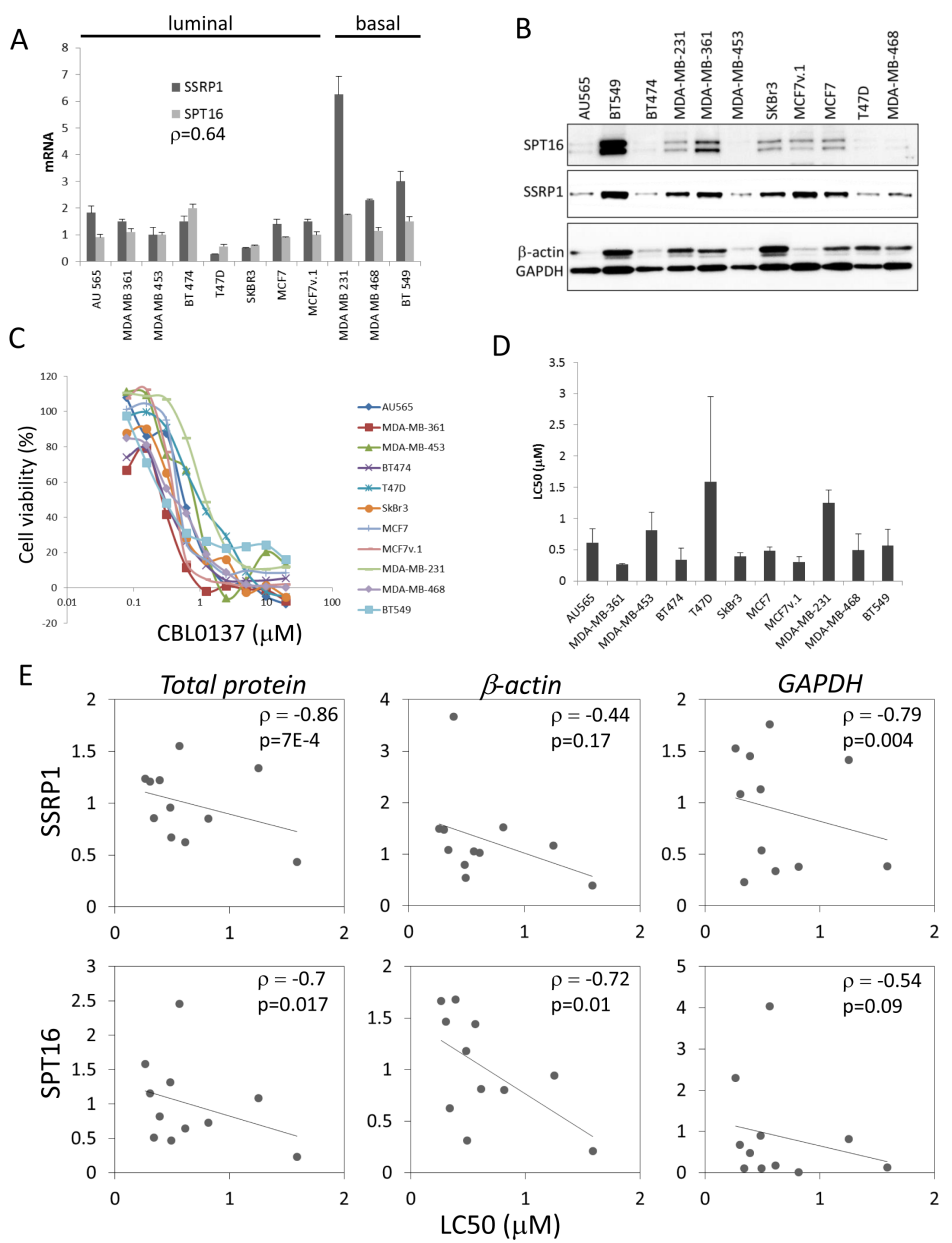
Correlation of FACT levels and cell sensitivity to CBL0137 in BrCa cells **A.** mRNA levels of SSRP1 and SPT16 in BrCa cells were assessed *via* RT-PCR and normalized to the level of GAPDH mRNA. Mean of three replicates +/− SD. p - Spearman correlation coefficient between SSRP1 and SPT16 expression. **B.** Western blotting of total cell lysates probed with the indicated antibodies. Equal amounts of protein were loaded for all cell lines. **C.** 72h cytotoxicity assay with CBL0137. Mean of three replicates in representative experiment. **D.** LC50 of CBL0137. Mean of all experiments (2-4 for different cell lines) +/− SD. **E.** Dot plots and linear trend lines between SSRP1 or SPT16 protein levels normalized *via* different means, total protein, beta-actin or GAPDH, indicated in italic above plots, and LC_50_ of CBL0137. Numbers in the upper right corner is Spearman correlation coefficients (ρ) and p-values.

Expression of both FACT subunits, SSRP1 and SPT16, is significantly elevated in BrCa samples *versus* normal mammary epithelial cells [1, 25]. However, there is significant variability in SSRP1 and SPT16 levels in clinical samples of BrCa [1], [2]. To understand the functional significance of high FACT expression, we compared the sensitivity of BrCa cells with different basal levels of FACT to FACT inhibition. To this end, we determined the SSRP1 and SPT16 mRNA and protein levels in several BrCa cell lines of different subtypes using RT-PCR and western blotting. Normalization of FACT levels across the panel of cell lines was performed using total mRNA or protein levels as well as levels of expression of housekeeping genes. To compare BrCa cell sensitivity to FACT inhibition, we treated cells with indirect FACT inhibitor, CBL0137 [21]. All tested BrCa cell lines, which expressed variable amounts of both FACT subunits, were sensitive to CBL0137 (Figure 1A-1D). We observed a negative correlation between the levels of both FACT subunits and the LC_50_ of CBL0137 regardless of the normalization method used (Figure 1E and Supplementary Table S1). Thus, we hypothesized that cells with higher basal FACT levels may be more sensitive to FACT inhibition than cells with lower basal FACT levels. Since CBL0137 is an indirect inhibitor of FACT and has FACT-independent activity [32], we proposed that use of more specific tools for FACT inactivation, such as shRNAs, would allow assessment of the sensitivity of the panel of cell lines to FACT inhibition more accurately.

**Figure 2:**
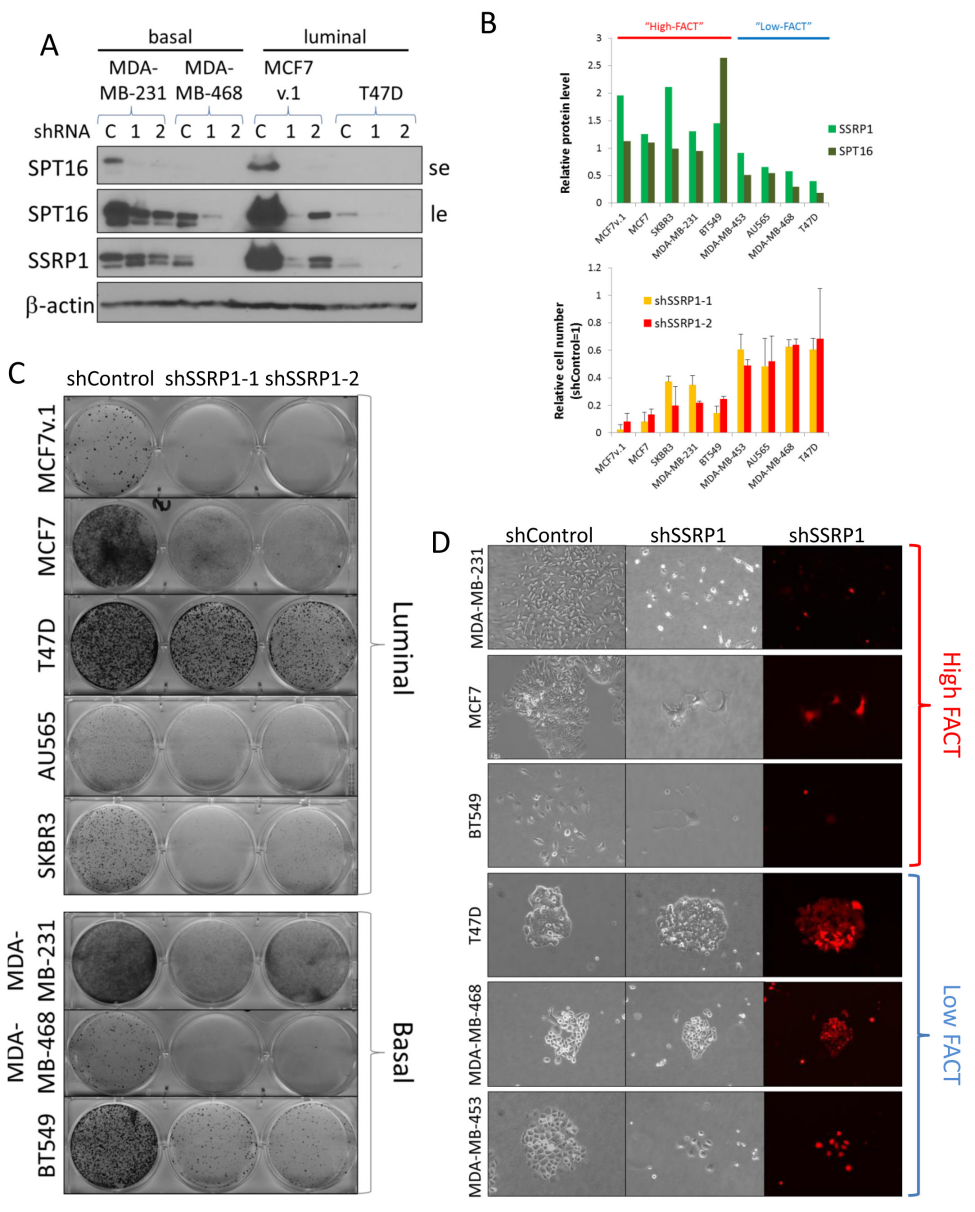
Sensitivity of BrCa cells to FACT knockdown **A.** Western blotting of BrCa cells transduced with either control shRNA (C) or two independent shRNAs to SSRP1 (1 and 2), probed with the indicated antibodies. SE - short exposure, LE - long exposure. **B.** Negative correlation between basal levels of FACT subunits in BrCa cells and sensitivity of the cells to FACT knockdown. Upper plot - relative amount of SSRP1 and SPT16 proteins assessed using ImageJ software. Average of three ways of normalization. Cells were arbitrary categorized as “high” and “low-FACT”. Lower plot - relative number of cells that survived puromycin selection after transduction with the same titer viruses with shRNAs to SSRP1 *versus* control shRNA (taken as 1). Mean of two replicates +/− SD. **C.** Photographs of plates transduced with the indicated lentiviral constructs at the same titer that survived puromycin selection and stained with methylene blue. **D.** Morphology of representative colonies formed upon transduction of cells with control shRNA or shRNA to SSRP1-2. Bright-field and fluorescent microscopy.

Importantly, as it was previously shown, stability of the two FACT protein subunits depends on their interaction and therefore knockdown of any one of the subunits leads to downregulation of the other [33]. The effect is specifically strong when shRNAs to SSRP1 are used. These shRNAs causes even faster reduction of the SPT16 subunit, than SSRP1, which is observed in some cell lines (e.g. MDA-MB-231, Figure 2A). In this study, we used two different shRNAs to the 3′UTR of SSRP1 as the most effective tools to inhibit FACT expression.

All BrCa cells tested were sensitive to FACT knockdown, but the degrees of sensitivity as well as the consequences of this downregulation were different between cell lines (Figure 2B-2D). Significant negative correlations between cells sensitivity to FACT knockdown and basal protein levels of SSRP1 (r = -0.67 (*p* = 0.05)) and SPT16 (r = -0.93 (*p* < 0.001)) were observed (Figure 2B, Supplementary Table S1), indicating that BrCa cells with higher expression of FACT are more dependent on it for growth and viability than cells with low levels of both subunits.

**Figure 3:**
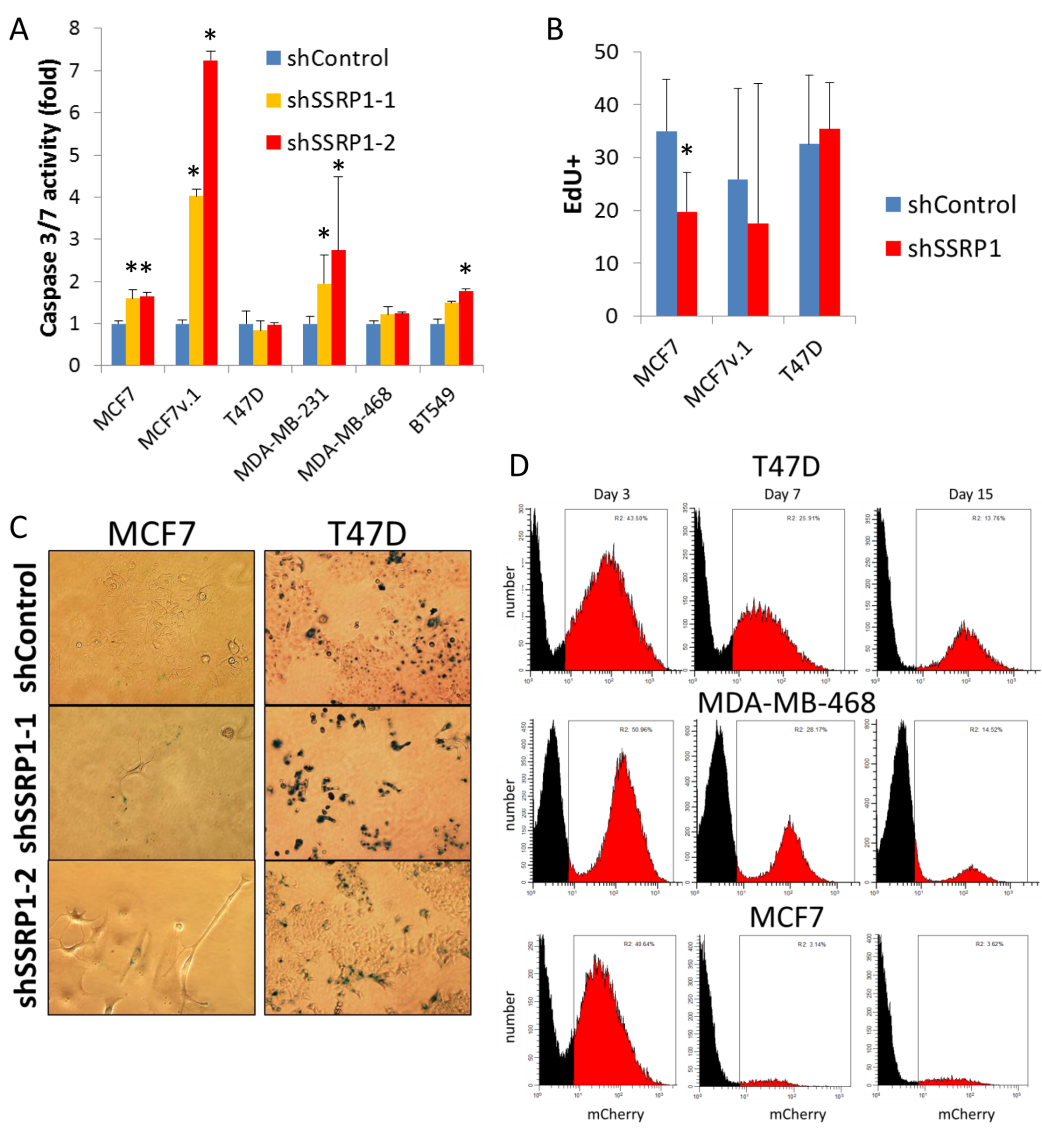
Consequences of FACT knockdown in BrCa cells **A.** and **B.** Level of caspase 3/7 activity (A) and EdU incorporation (B) in cells transduced with control shRNA or shRNAs to SSRP1 72 hours after transduction. Mean of three replicates +/− SD, * - *p* < 0.05. **C.** Acidic beta-galactosidase staining of cells transduced with the indicated shRNAs that survived puromycin selection. **D.** Proportion of mCherry positive cell (red) in cell populations transduced with shRNA to SSRP1 at different time points after transduction without puromycin selection assessed using flow cytometry.

The consequences of FACT knockdown did not depend on BrCa subtype (Figure 2C). Reduction of FACT led to visible cell death in some luminal (MCF7v.1) and basal (MDA-MB-231) cell lines whereas other cell lines were either growth inhibited (luminal MCF7, basal BT549), or continued to grow, albeit slower than control cells (luminal T47D and basal MDA-MB-468) (Figure 3). Death of cells was confirmed by caspase activation (Figure 3A) and growth arrest by reduced EdU incorporation (Figure 3B). Some growth arrested cells (e.g. MCF7) were enlarged, flattened and formed dendritic-like protrusions (Figure 2B and Supplementary Figure S1), which was suggestive of senescence, however, this was not confirmed by acidic beta-galactosidase staining (Figure 3C).

We used a pair of syngeneic MCF7 cells that innately differed in their basal level of FACT, one obtained from ATCC (MCF7) and one from the lab of Dr. Bacus [34] (MCF7v.1). The close origin of the two cell lines was confirmed with short tandem repeat analysis (92% identical), however there were certain differences in the profiles of gene expression (Supplementary Table S2). Cells with very high FACT expression (MCF7v.1) did not tolerate FACT inhibition at all and died *via* apoptosis. In contrast, cells with a moderate level of FACT (MCF7) were growth arrested (Figure 3A-3C). Interestingly, the two cell lines with the lowest basal FACT expression, luminal T47D and basal MDA-MB-468, continued to proliferate (Figure 2D, note visible red colonies, and Figure 3C-3E), although they produced less colonies upon transduction with SSRP1 shRNAs than control shRNA (Figure 2B, 2C). We did not detect any difference in EdU incorporation of T47D or MDA-MB468 cells with and without FACT knockdown (Figure 3B). However, passaging of these cells led to the gradual reduction in the proportion of shRNA transduced cells (assessed *via* expression of mCherry marker present in shRNA vector) in shSSRP1 but not shControl cultures (Figure 3D). There was also no caspase activation or increase in acidic beta-galactosidase staining cells in these two cell lines upon reduction of FACT levels (Figure 3A, 3C), suggesting a slowing down of the cell cycle rather than death or growth arrest in the majority of these cell lines. Thus, we observed that cells with the lowest basal level of FACT expression are the least sensitive to FACT inhibition, which is important to understand for further use of anti-FACT therapy in the clinic.

### Inhibition of FACT does not change the rate of general transcription

**Figure 4:**
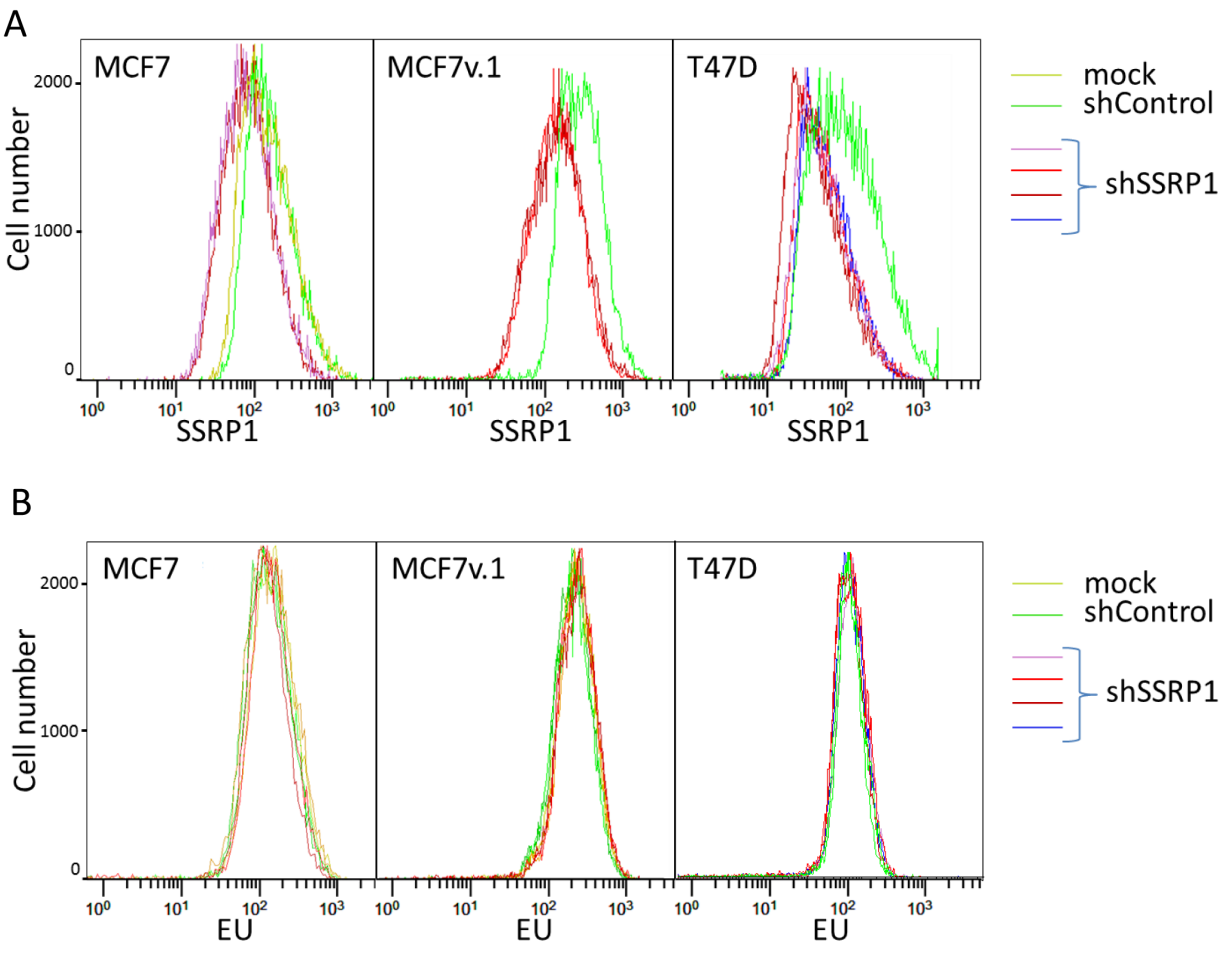
Effect of FACT knockdown on the rate of transcription in BrCa cells **A.** Flow cytometric analysis of SSRP1 level in BrCa cells transduced with different shRNAs or untransduced (mock) 72 hours after transduction. **B.** EU incorporation into cells shown on panel A. Positive (Actinomycin D) and negative (cells with no EU added) controls are shown on Supplementary Figure S2.

The most well established function of FACT is regulation of transcription through the maintenance of chromatin organization [35]. Therefore, a functional deficit of FACT may lead to a change in the rate of transcription. To test this hypothesis, we compared RNA synthesis in several cell lines before and after FACT knockdown using 5-Ethynyl Uridine (EU). For these analyses, we selected three BrCa cell lines of luminal subtype for which the response to FACT reduction was significantly different: MCF7v.1, which die *via* apoptosis, MCF7, which undergo growth arrest, and T47D cells, which slightly decelerate proliferation. In these studies, we measured the incorporation of EU into newly synthesized RNA 72 hours after transduction with control or SSRP1 shRNA. In parallel, we determined the SSRP1 protein level present in each cell line using flow cytometry. In all three cell lines, we observed a negative shift in SSRP1-associated fluorescence in shSSRP1-transduced cells relative to control cells, however, there was no shift in EU incorporation (Figure 4A and 4B, Supplementary Figure S2A). Furthermore, when cells were incubated with EU for different periods of time, including a very short incubation of 15 minutes that would detect more subtle differences in the rate of RNA syntheses, we did not observe any significant change in EU incorporation upon FACT knockdown (Figure 4 and Supplementary Figure S2B).

### Cell specific role of FACT in regulation of gene expression

**Figure 5:**
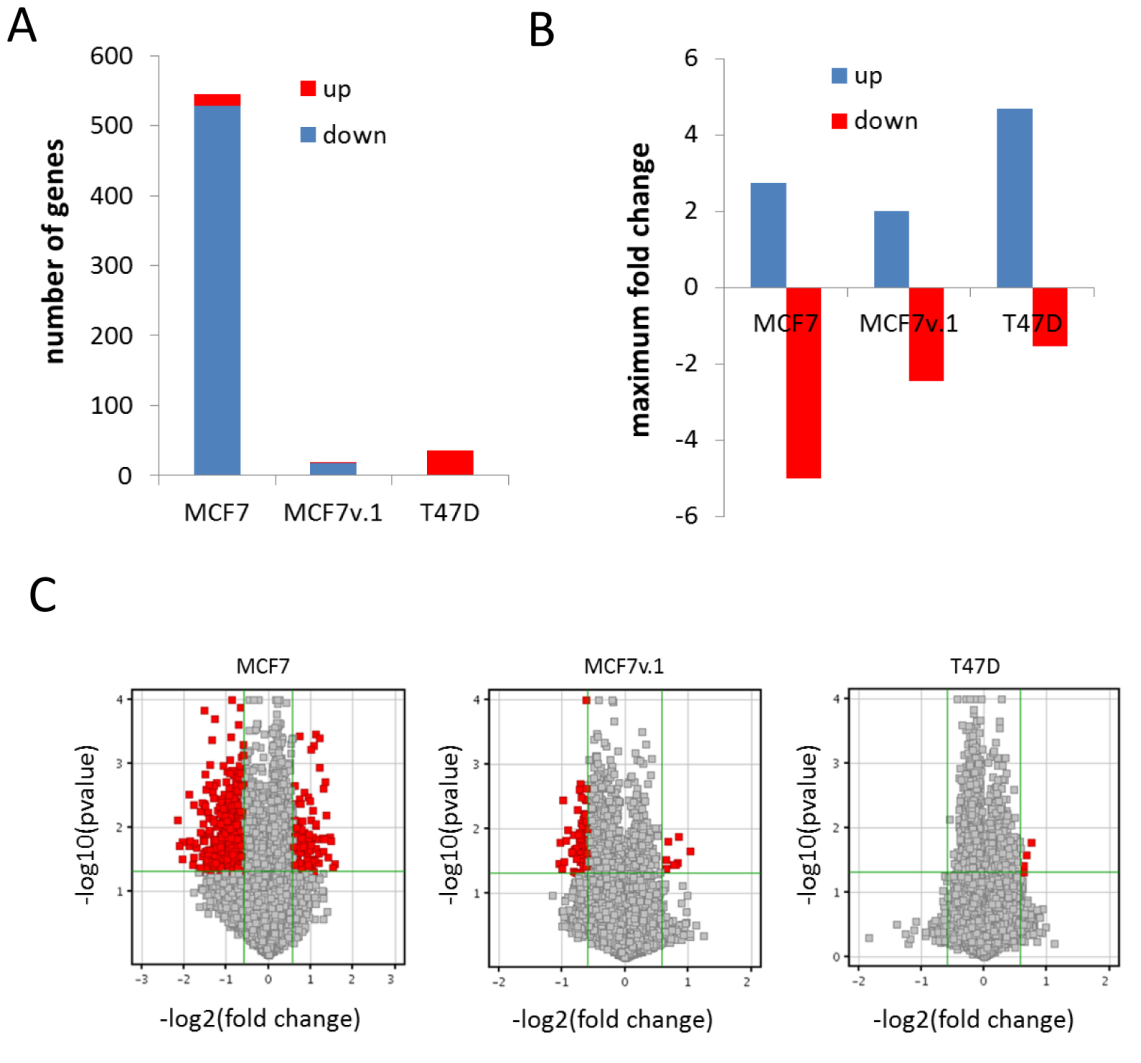
Analysis of gene expression changes in BrCa cells upon FACT knockdown **A.** Number of genes with expression significantly increased or decreased (2 fold, *p* < 0.05) 72h after transduction with shRNAs to SSRP1 *versus* control shRNA. **B.** Average fold change in expression of all down or up-regulated genes 72h after transduction with shRNAs to SSRP1 *versus* control shRNA. **C.** Volcano plots of gene expression changes in three cell lines with red dots showing significantly changed genes (2 fold, *p* < 0.05) among all genes (grey dots).

To determine if knockdown of FACT altered expression of individual genes, we analyzed gene expression changes upon FACT downregulation in the same three BrCa cell lines 72 hours after transduction with two different shRNAs to SSRP1 and control shRNA, when reduction of the protein levels of FACT subunits was evident, but before any dramatic decrease in cell number was observed (Supplementary Figure S3A, S3B). Analyses of array hybridization demonstrated that the maximum number of changes, including the number of affected genes and fold change in expression, were observed with MCF7 cells, which undergo growth arrest in response to FACT knockdown. Minimal changes were observed with T47D, although expression of FACT subunits was significantly inhibited in these cells (Supplementary Figure S3A). This was consistent with observing only a minor phenotypic response to FACT downregulation in T47D cells (Figure 5A-5C and Supplementary Figure S3C, S3D). Intermediate changes in gene expression were observed for MCF7v.1 cells, which undergo apoptosis, most probably due to the quick death of cells with the most effective knockdown or the death of those cells occurring without considerable changes in gene expression (Figure 5A-5C). Alternatively, the level of inhibition of FACT subunits expression may be not enough (Supplementary Figure S3A), but infection of MCF7v.1 cells with more concentrated shSSRP1 virus led to cell death.

**Figure 6:**
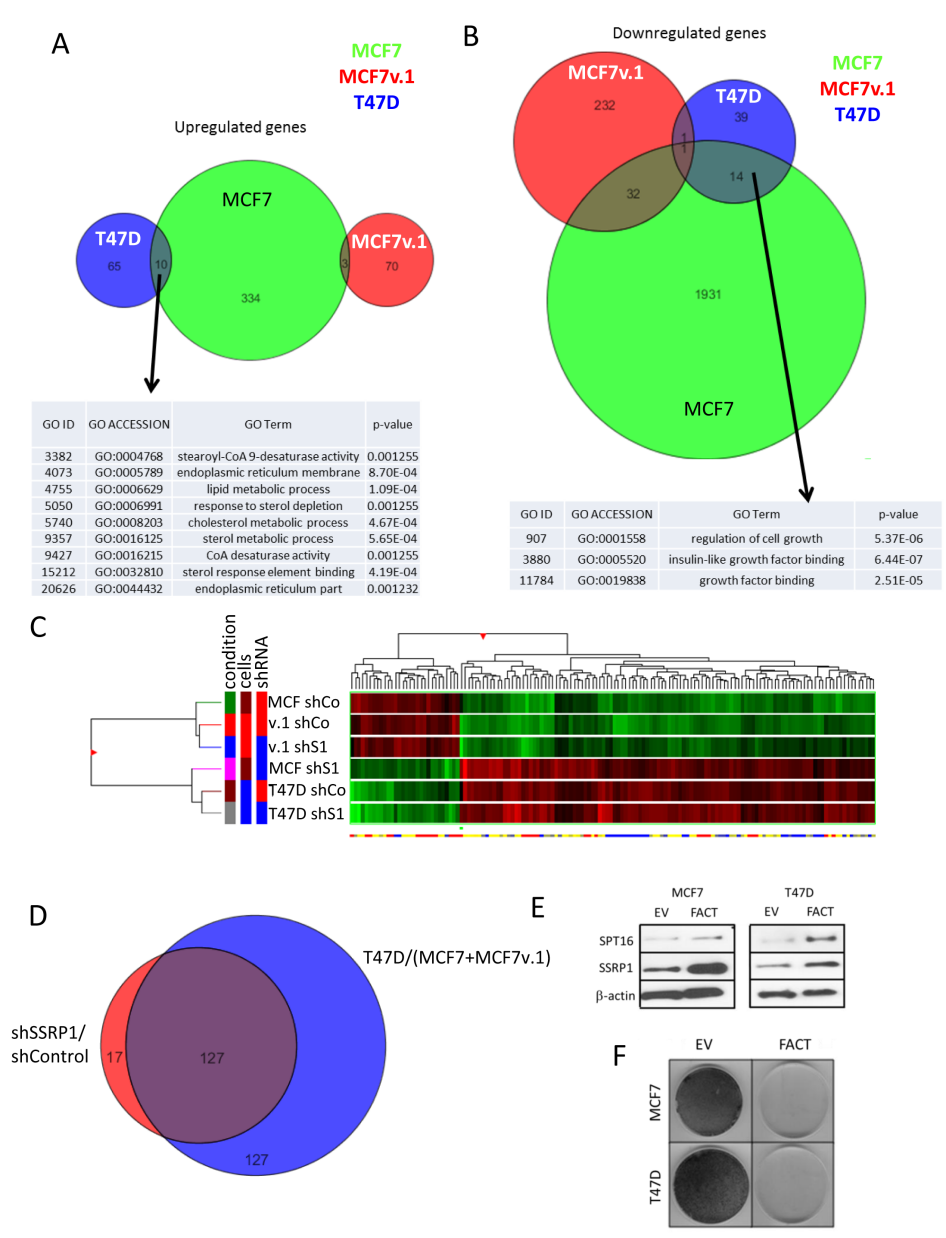
Changes in transcriptional programs in BrCa cells upon FACT knockdown **A.** and **B.** Venn diagrams of genes up- (A) or down-regulated (B) in three BrCa cell lines following FACT knockdown and GO analyses of the commonly regulated genes. **C.** Similarity between different samples assessed via unsupervised hierarchical clustering of samples and genes using Euclidian distance metric. Dendrogram and heatmap of gene expression with red - high and green - low level of gene expression across samples. **D.** Venn diagram of genes with expression that is different between MCF7+MCF7v.1 and T47D cells and MCF7 cells before and after FACT knockdown. **E.**-**F.** Overexpression of FACT is toxic for BrCa cells. Western blotting E. and methylene blue staining F. of cells co-transduced with SSRP1 and SPT16 expression constructs (FACT) or two empty vectors followed by selection with two antibiotics. Cells for western were collected 48 hours after transduction and before antibiotic selection.

There were significantly more genes for which expression was inhibited by FACT knockdown than genes for which expression was induced in both variants of MCF7 cells (Figure 5B, 5C). This distribution was reversed in T47D cells (Figure 5B, 5C). There was no significant overlap among genes for which expression was changed following SSRP1 shRNA transduction in the three cell lines. However, genes that were upregulated > 2 fold in MCF7 and T47D cells were classified by GO terms as being involved in lipid and steroid metabolism (Figure 6A), which is a characteristic of differentiated mammary epithelial cells. Genes that were downregulated ( > 2 fold) in the same cell lines belonged to the category “Control of cell growth” (Figure 6B). Based on these findings, we proposed that FACT has a cell-specific role in regulating the expression of individual genes, but generally negatively regulates expression of genes involved in the differentiation of BrCa luminal cells and supports transcription of genes involved in cells growth.

The most interesting changes in the pattern of gene expression following SSRP1 shRNA treatment were observed when we performed unsupervised hierarchical clustering of all samples. FACT knockdown with any of shRNAs to SSRP1 in MCF7 cells made the cells remarkably similar to T47D cells (Figure 6C). To confirm this observation, we selected all genes that were different between T47D and two MCF7 cell lines (fold > 2 d, *p* < 0.05) and compared this gene list with those that were changed in MCF7 cells in response to FACT knockdown (fold > 1.5, *p* < 0.05). These gene lists were almost identical (Figure 6D), suggesting that inhibition of FACT in both MCF7 cell lines converts their gene expression profile to closely resemble that of T47D cells. Thus, we found that two cell lines, initially selected based on their different levels of FACT, become very similar when we artificially equalize FACT expression, suggesting the role of FACT in defining the transcriptional landscape of BrCa cells. Interestingly, when we tried to determine whether an increase in FACT levels in T47D cells *via* transduction of SSRP1 and SPT16 expression constructs, would cause the cells to become MCF7-like, no cells survived (Figure 6E, 6F). A similar phenomenon occurred with other tumor cells when we tried to overexpress FACT subunits (Supplementary Figure S4). Since FACT level is increased during the process of oncogenic transformation [36] [1], the reason for toxicity of artificial FACT elevation is unclear and requires additional investigation.

To annotate any common functional characteristics of genes whose expression correlates with FACT level, we ran a Gene Set Enrichment Analysis (GSEA). GSEA demonstrated that genes downregulated upon FACT knockdown belong to four functional groups, (i) genes involved in regulation of cell cycle and DNA replication, (ii) genes overexpressed in ES cells or in undifferentiated *versus* differentiated cancers; (iii) genes upregulated by several pro-oncogenic factors, including Myc, EGFR, RalA, RalB, and RhoA, and (iv) genes that are targets of transcriptional factors of the E2F family (Table 1).

**Table 1:** GSEA of genes which expression depends on high FACT level

Functional group	Gene Set Name	# Genes in Gene Set	Description	# Genes in Overlap (k)	k/K	p-value	FDR q- value
DNA replication, proliferation, cell cycle	CHANG_CYCLING_GENES	148	Fibroblast serum response genes showing periodic expression during the cell cycle; excluded from the core serum response signature.	49	0.3311	2.04E-88	2.40E-85
	BENPORATH_CYCLING_ GENES	648	Genes showing cell-cycle stage-specific expression [PMID=12058064].	60	0.0926	7.85E-73	5.30E-70
	REACTOME_CELL_CYCLE_ MITOTIC	325	Genes involved in Cell Cycle, Mitotic	40	0.1231	1.07E-52	1.73E-50
	BENPORATH_ PROLIFERATION	147	Set 'Proliferation Cluster': genes defined in human breast tumor expression data.	32	0.2177	1.46E-50	2.09E-48
	REACTOME_CELL_CYCLE	421	Genes involved in Cell Cycle	41	0.0974	1.01E-49	1.25E-47
	WHITFIELD_CELL_CYCLE_ LITERATURE	44	A list of known cell cycle regulated genes that was compiled from the literature by the authors.	21	0.4773	6.68E-42	6.18E-40
	REACTOME_MITOTIC_G1_ G1_S_PHASES	137	Genes involved in Mitotic G1-G1/S phases	18	0.1314	1.95E-24	9.68E-23
	WHITFIELD_CELL_CYCLE_ G2	182	Genes periodically expressed in synchronized HeLa cells (cervical carcinoma), with peak during the G2 phase of cell cycle.	19	0.1044	9.03E-24	4.35E-22
	REACTOME_ DNA_REPLICATION	192	Genes involved in DNA Replication	24	0.125	9.16E-32	5.93E-30
E2F targets	MARSON_BOUND_BY_ E2F4_UNSTIMULATED	728	Genes with promoters bound by E2F4 [GeneID=1874] in unstimulated hybridoma cells.	56	0.0769	5.03E-63	1.32E-60
	KONG_E2F3_TARGETS	97	Genes up-regulated in MEF cells (embryonic fibroblasts) at 16 hr after serum stimulation and knockdown of E2F3 [GeneID=1871] by RNAi.	23	0.2371	2.23E-37	1.78E-35
	ISHIDA_E2F_TARGETS	53	Genes up-regulated in MEF cells (embryonic fibroblast) after expression of E2F1 or E2F2 [GeneID=1869;1870].	18	0.3396	1.11E-32	7.48E-31
Undifferentiated cell state	BENPORATH_ES_1	379	Set 'ES exp1′: genes overexpressed in human embryonic stem cells according to 5 or more out of 20 profiling studies.	34	0.0897	7.51E-40	6.57E-38
	SARRIO_EPITHELIAL_ MESENCHYMAL_ TRANSITION_UP	180	Genes up-regulated in MCF10A cells (breast cancer) grown at low (mesenchymal phenotype) compared to those grown at high (epithelial, basal-like phenotype) confluency.	34	0.1889	1.97E-51	3.10E-49
	WONG_EMBRYONIC_ STEM_CELL_CORE	335	The 'core ESC-like gene module': genes coordinately up- regulated in a compendium of mouse embryonic stem cells (ESC) which are shared with the human ESC-like module.	31	0.0925	9.57E-37	7.53E-35
	RHODES_UNDIFFERENTIA TED_CANCER	69	Genes commonly up-regulated in undifferentiated cancer relative to well-differentiated cancer, based on the meta-analysis of the OncoMine gene expression database.	20	0.2899	1.36E-34	1.02E-32
Upregulated in response to oncogenes	WEI_MYCN_TARGETS_ WITH_E_BOX	795	Genes whose promoters contain E-box motifs and whose expression changed in MYCN-3 cells (neuroblastoma) upon induction of MYCN [GeneID=4613].	35	0.044	2.91E-30	1.81E-28
	YU_MYC_TARGETS_UP	42	Genes up-regulated in B cell lymphoma tumors expressing an activated form of MYC [GeneID=4609].	15	0.3571	8.57E-28	5.12E-26
	OXFORD_RALA_OR_RALB _TARGETS_UP	48	Genes up-regulated after knockdown of RALA or RALB [GeneiD=5898;5899], which were also differentially expressed in bladder cancer compared to normal bladder urothelium tissue.	14	0.2917	1.49E-24	7.48E-23
	KOBAYASHI_EGFR_ SIGNALING_24HR_DN	251	Genes down-regulated in H1975 cells (non-small cell lung cancer, NSCLC) resistant to gefitinib [PubChem=123631] after treatment with EGFR inhibitor CL-387785 [PubChem=2776] for 24h.	71	0.2829	5.70E-125	2.69E-121
	BERENJENO_ TRANSFORMED_ BY_RHOA_UP	536	Genes up-regulated in NIH3T3 cells (fibroblasts) transformed by expression of contitutively active (Q63L) form of RHOA [GeneID=387] off plasmid vector.	54	0.1007	4.18E-67	1.64E-64

**Figure 7:**
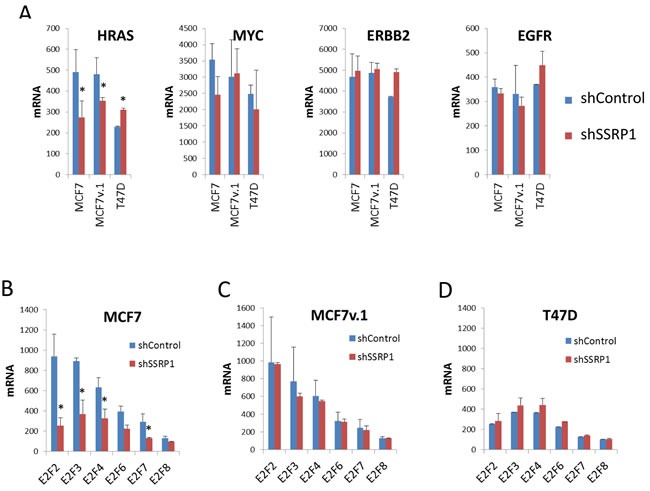
Effect of FACT knockdown on the mRNA levels of proto-oncogenes commonly involved in BrCa A. and members of E2F family of transcription factors B.-D. Mean normalized signal intensity of two replicates from microarray hybridization. Error bars - SD. * - *p* < 0.05.

Among oncogenes known to be involved in mammary epithelial tumorigenesis, such as H-RAS, ERBB2, EGFR, and MYC, only expression of H-RAS mRNA was significantly higher in MCF7 cell variants than T47D cells and was also reduced upon FACT knockdown (Figure 7A). MYC, EGFR and ERBB2 mRNA were expressed in all cell variants at similar levels and were not changed upon FACT knockdown (Figure 7A). Expression of members of the E2F family of transcription factors was higher in both MCF7 cell variants than in T47D cells, and was reduced upon FACT knockdown in MCF7 cells (Figure 7B-7D).

Because of this, we hypothesized that “high” FACT cells may divide faster than “low” FACT cells. They also may have higher proportion of cells with cancer stem cell (CSC) properties.

### FACT level correlates with proliferation rate and presence of CSC in BrCa cell lines

**Figure 8:**
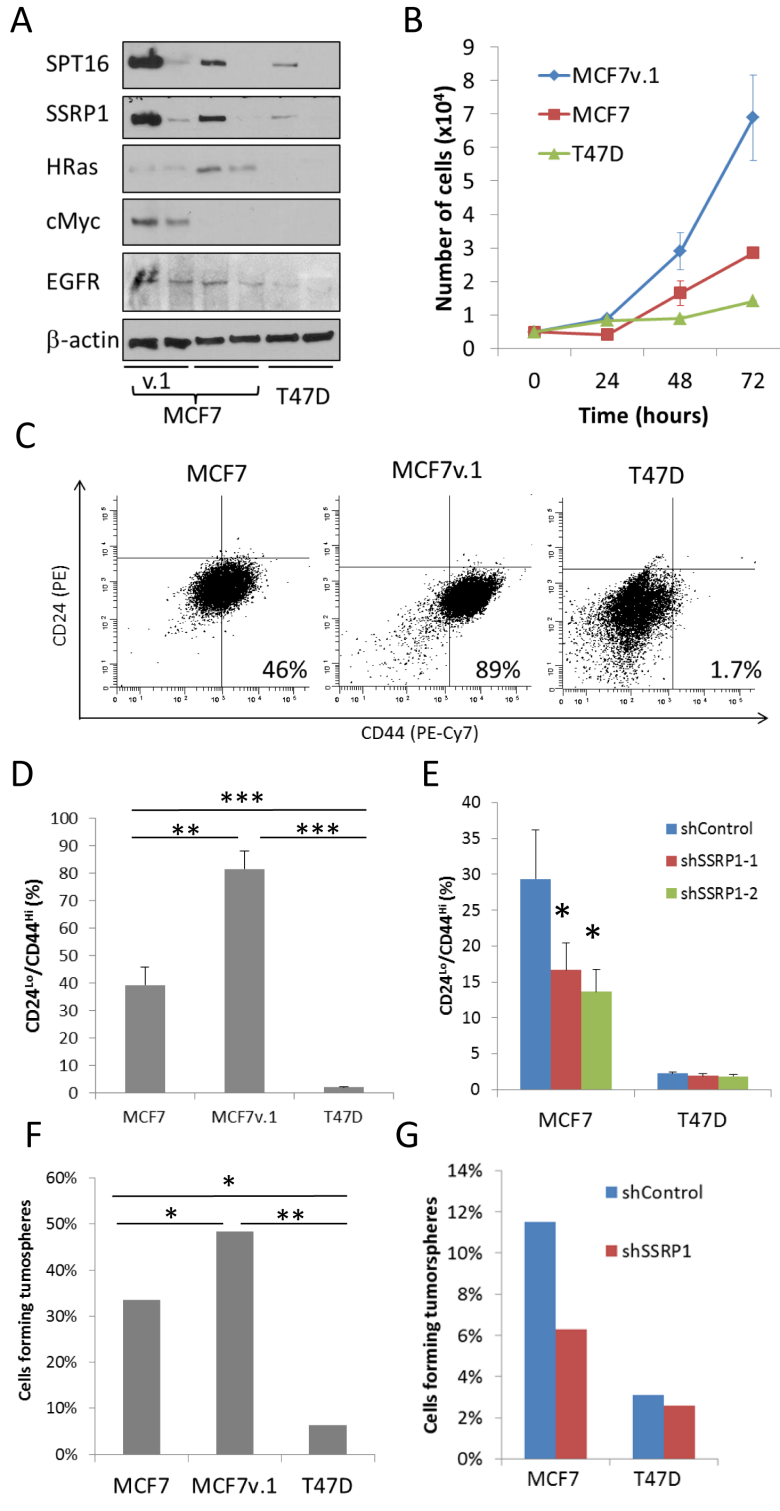
Phenotypical differences between cells with high and low FACT expression **A.** Western blotting of extracts of cell transduced with control shRNA or shSSRP1-1 72 hours before lysis probed with indicated antibodies. **B.** Comparison of growth of three BrCa cell lines *via* direct cell counting. Mean of three replicates +/− SD from representative experiment. **C.**-**D.** Flow cytometric analysis of CD24 and CD44 expression in three BrCa cells lines. Data of representative experiment **C.** and mean of three experiments + SD **D.** Gating was done using cells stained with control isotype matching antibodies (vertical and horizontal lines on C). **E.** Change in CD24/CD44 staining in MCF7 and T47D cells transduced with either control shRNA or shSSRP1-1. Mean of 2 experiments + SD. **F.**-**G.** Tumorsphere formation assay with three BrCa cell lines (F) or MCF7 and T47D cells transduced with control shRNA or shRNAs to SSRP1 (G). Proportion of wells with tumorspheres in 384 well plate in which one cell per well was plated (see details in Material and Methods). * - *p* < 0.05, ** - *p*< 0.01, *** *p* < 0.001.

To evaluate whether FACT-dependent differences in gene expression are translated into differences in biological properties of the cells, we compared the protein levels of several proto-oncogenes in the three luminal BrCa cell lines with and without FACT knockdown using western blotting. H-Ras expression was the highest in MCF7 cells and the lowest in T47D cells, and was reduced upon FACT knockdown (Figure 8A). Surprisingly, and in contrast to what was observed at the mRNA level, c-Myc protein level was the highest in MCF7v.1 cells and was reduced upon FACT knockdown (Figure 8A). This suggests that there is some connection between FACT and c-Myc on the protein level and explains why genes expressed at a higher level in “high FACT” cells compared to in “low FACT” cells belong to the category of Myc targets (Table 1). EGFR protein levels was also higher in both MCF7 cell lines than in T47D and was reduced upon FACT knockdown in MCF7 and T47D cells (Figure 8A).

As predicted by gene expression, analysis of the population doubling times of the three BrCa cell lines showed a negative correlation with FACT levels (MCF7v.1 < MCF7 < T47D) (Figure 8B). In contrast, a positive correlation was observed between FACT level and the proportion of CD24^low^/CD44^high^ cells, an established phenotype of BrCa stem cells [37] (Figure 8C, 8D). The proportion of CD24^low^/CD44^high^ cells was reduced upon transduction with shRNAs to SSRP1 (Figure 8E). To compare functional properties of CSC, we tested the ability of cells to form tumorspheres in 3D serum-free conditions, which perfectly correlated with FACT levels in the three BrCa cell lines tested (MCF7v.1 > MCF7 > T47D). Furthermore, knockdown of FACT cells substantially reduced this property in MCF7 cells, but not in T47D cells. (Figure 8F, 8G).

## DISCUSSION

BrCa is a very diverse set of diseases in which progression may be dramatically different, varying from indolent cases that require almost no treatment to highly aggressive lethal disease. Although several prognostic markers are available, they do not accurately predict BrCa progression. Moreover, even if additional markers were currently available, treatment options are still limited since aggressive chemotherapy is not completely justified at an early stage due to the harmful effect of these therapies on healthy tissues. Therefore, an ideal marker of aggressive cancer should be a factor that plays a major role in conferring tumor aggressiveness and, therefore, may also be used as a target for the treatment of this cancer.

Current anti-cancer drug discovery is focused on the identification of small molecules targeting mutated oncogenes. This approach has had limited success due to inter-tumor and intra-tumor heterogeneity [38] as well as the high redundancy of signaling pathways in cells [39]. However, some cellular processes that are essential for sustained tumor growth and viability are uniformly activated in tumor cells, e.g. DNA replication. Targeting of DNA replication is not popular anymore due to insufficient specificity of this approach and induction of DNA damage. Here, we propose that there are other critical cellular processes that are almost universally active in tumor cells but much less essential for normal cells, which can be targeted for the purpose of cancer treatment. The activity of certain histone chaperones is one such process [5–9]. Histone chaperones were traditionally viewed as ubiquitously expressed housekeeping factors, however, the accumulation of recent studies shows that this is not the case [5–9] (e.g., spatial and temporal cell-specific expression of FACT [22, 25, 40]). FACT is practically never mutated in cancer (cBioPortal), but almost always elevated [1, 2, 40, 41]. Early embryonic lethality (3.5dpc) caused by SSRP1 loss in mice [42] and toxicity of spt16 and pob3 (yeast SSRP1 homologue) mutations in yeast [43] demonstrated the principal necessity of FACT for general transcription and/or replication. However, recent findings suggest that FACT is essential for only certain types of transcription or replication in some classes of cells [1, 22–24]. Tumor cells with low and high basal levels of FACT are both viable, but differ in the transcriptional profiles and biological markers associated with tumor aggressiveness. Importantly, our data imply that when FACT is present at a low level in tumor cells, a differentiation-related transcriptional program is mostly inhibited, whereas a higher level of FACT accelerates tumor cell proliferation. Moreover, tumor cells expressing high levels of FACT are more addicted to FACT function and cannot survive its inhibition.

The presence of “high FACT” is associated with the transcription of genes that regulate cell cycle progression and the maintenance of the undifferentiated cell state. Our study and related literature demonstrated that two cell lines, MCF7 and T47D, established from very similar samples (i.e., pleural effusion of invasive breast carcinoma) and both belonging to the luminal A subtype based on marker expression, have quite different biological properties, including proliferation rate [44], expression of CSC markers [45], capability for clonal anchorage independent growth [45], and formation of primary and metastatic tumors in mice [44], [46]. Based on all these parameters, MCF7 cells are more aggressive than T47D. Not surprisingly, the gene expression profiles of these cells are quite different and clearly reflect these biological differences. Inhibition of FACT in these two cell lines have different consequences with minimal changes in transcription and phenotype for “low FACT” T47D cells, but induction of death in cells with a highest level of FACT (MCF7v.1) or profound growth arrest in cells with a slightly lower level of FACT (original MCF7). Strikingly, inhibition of FACT made the gene expression profile of MCF7 cells very similar to that of T47D.

FACT is not a classic DNA binding transcription factor or signal messenger. It does not have defined target genes or a clear mechanism to selectively affect expression of a subset of genes. At the same time, FACT is not a general transcription factor, because there is a definite selectivity in FACT genome wide localization [1], and in gene expression changes resulting from FACT knockdown [47]. Here, we show that this selectivity is cell dependent, even among cells of very close origin. We propose that cell specific transcription factors control gene expression in BrCa cells. FACT is needed to make this transcriptional program effective, since a reduction in the FACT level results in abrogation of expression of large sets of genes. Importantly, the degree of changes of individual genes is very modest, at maximum 30-40%. This is in line with the biochemical function of FACT: RNA polymerases can in principle transcribe *in vitro* through a chromatinized template even without FACT. However, FACT presence diminishes polymerase pausing and, therefore, increases the efficiency of transcription [19]. This modest effect is not unique to FACT. The same was observed when another histone chaperone, Asf1b, was depleted from BrCa cells [9]. Thus, we propose that FACT as well as some other histone chaperones may be needed to provide “acceleration” of certain transcriptional programs above a threshold at which tumor aggressiveness is heightened.

FACT may also exert its effect on transcription of a selected pool of genes *via* its predominant effect on the transcription of some transcription factors, e.g. MYC. There are already several examples of a functional relationship between FACT and MYC proto-oncogene, such that high enrichment of FACT over the body of the MYC gene in chromatin, feed forward interaction between FACT and NMYC [40] in neuroblastoma and potential direct interaction of SSRP1 and c-Myc [48]. However, in our study we observed the strongest effect of FACT on the gene transcription in cells with undetectable level of c-Myc expression (MCF7, Figure 8A).

An alternative mechanism is that FACT plays a major role not in transcription, but in replication, such that decreased FACT levels leads to a slowing down of the cell cycle with a concomitant change in expression of all cell cycle dependent genes, including proto-oncogenes and E2F family. This scenario is possible, since a role for FACT in DNA replication has been demonstrated in different models [12, 29, 49]. This seems less probable than the former, however, because the response to inhibition of FACT in this case would be more uniform in different cell lines with a dose dependence to growth retardation whereas we observed apoptosis in high FACT cells and almost no response in low FACT cells. In addition, this mechanism also does not explain the change in expression of CSC markers upon FACT inhibition.

Importantly, clinical samples showed a higher correlation of FACT level with grade than stage of disease [1], [2]. This means that the proportion of “high FACT” tumors does not change significantly with disease development and therefore “high FACT” is observed even in patients with early stage disease. Thus, we propose that the predominant effect of targeting FACT will be the elimination of FACT positive BrCa cells with high malignant potential, which may be a way to combat the aggressiveness of early stage cases and, at the same time, be a safe alternative to current therapies since FACT is expressed in a very limited number of normal cells.

## MATERIALS AND METHODS

### Cells and reagents

MCF7, AU565, T47D, MDA-MB-231, -361, -453, -468, BT474, BT549 and SCBR 3 cells were obtained from ATCC. All cells were frozen and used at low passage number ( < 7). MCFv.1 cells were obtained from Sarah Bacus lab (Ventana Medical Systems, Inc., Westmont, IL, USA). Identity of cells was confirmed *via* short tandem repeat (STR) analyses done in the Genomics Core of RPCI (Buffalo, NY, USA). CBL0137 was provided by Incuron, LLC (Buffalo, NY, USA). Actinomycin D was purchased from Sigma-Aldrich (St. Louis, MO, USA). shRNAs to 3′UTR of SSRP1 and control shRNA (scrambled) were synthesized and cloned into lentiviral vector by GeneCopoeia (Rockville, MD, USA). Target sequences for shSSRP1-1: gtccctggattctgtgcca, for shSSRP1-2: cagtggggagacgtctta. pLV-SSRP1-neo, pLV-SPT16-hygro and corresponding control vectors were previously described [33].

Antibodies were used for immunoblotting (IB) or flow cytometry (FC) at the following titers: SSRP1 10D1 (Biolegend, San Diego, CA, USA), IB - 1:4000, FC - 1:200, SPT16 8D2 (Biolegend), IB - 1:1000; β-actin AC15 (Sigma-Aldrich, St. Louis, MO, USA), IB - 1:20000; H-Ras C20 (Santa Cruz Biotechnology, Santa Cruz, CA, USA), IB - 1:200; cMyc (Santa Cruz Biotechnology), IB - 1:200, PE Mouse Anti-Human CD24 Clone ML5 (BD Pharmingen, San Diego, CA, USA), FC - 1:50, CD44-PE-Cy™7 Mouse Anti-Human CD44 Clone G44-26 (also known as C26) (BD Pharmingen), FC - 1:50, IgG PE-Cy7 and IgG PE were also from BD Pharmingen, FC - 1:50. HRP-conjugated secondary antibodies were from Santa Cruz Biotechnology.

### Transfection

It was done using Lipofectamine 2000 (Invitrogen, Grand Island, NY, USA) or Polyjet transfection reagent (SignaGen Lab, Rockville, MD, USA) according to manufacturer instructions.

### Lentiviral transduction

Lentiviral packaging was performed as previously described [50]. Virus was concentrated using Amicon Ultra-4 centrifugal filter units (Millipore, Billerica, MA, USA). Virus titer was determined *via* titration of virus on HeLa cells followed by detection of the proportion of infected cells *via* fluorescent activated cell sorting based on the detection of mCherry expressed from an independent promoter in the same vector as shRNA.

### Cytotoxicity assay

was done as already described [21]. Briefly, cells were plated in 96 well plates at a density that allowed 72 hours growth of untreated cells (5-10×10^3^ cell per well). Cells were treated with 10 serial dilutions of CBL0137 for 24 hours, after which medium was changed to drug-free. 50μm of 9-aminoacridine was used as a positive control for complete cell death. Cell viability was detected at 72 hours after start of treatment using Cell Titer Blue assay (Promega, Madison, WI, USA). Experiments were run in triplicate and repeated at least twice.

### Colony formation assay

The same titer of control and SSRP1 targeting lent viruses was used to infect cells at 1TU/cell (as defined on HeLa cells). One control and two different shRNAs construct to SSRP1 were used. Forty-eight hours after transduction, cells were selected in the presence of 1-2μg/ml puromycin for 3 days. After selection, cells were maintained in drug free medium until visible colonies were formed in shControl plates. Colonies were stained with methylene blue and photographed. Quantitation was done *via* spectrophotometry of methylene blue solution extracted with 1% SDS using PerkinElmer^®^ Multimode Plate Reader VICTOR™ X3 (Perkin Elmer, Waltham, MA, USA). Experiments were repeated at least twice with 2 replicates in each.

### Tumorsphere formation assay

was done as described previously [51] with one modification. Instead of 96 well plates, cells were plated in black, clear round bottom 384 well spheroid microplates with Ultra-Low Attachment surface (Corning, Corning, NY, USA) at 1 cell per well using MicroFlo Select cell dispenser (BioTek, Winooski, VT, USA). Experiment was repeated two times.

### Semi-quantitative reverse transcription PCR

was performed using PCR Master Mix (Promega) on iCycler (BioRad, Hercules, CA, USA) with the following primers: SPT16 (Fw: ctagggtttgggatgggaat; Rv: gattgtggcaatgtgaaacg; Tm 58C), SSRP1 (Fw: cgtggtcgttatgacattcg; Rv: ttcatgccctctttcaatcc; Tm 58C), GAPDH (Fw: ggcttccgtgtccccactgc; Rv: ggctggtggtccaggggtct, Tm 60C). Experiment was repeated two times.

### Western blotting

was done using a standard protocol. Total cell lysates were prepared using 1xCell Culture Lysis reagent (CCLR) (Promega) supplied with Protease Inhibitor cocktail (Roche, Basel, Switzerland). Lysates were left on ice for 30min- vortexing every 10min followed by sonication twice for 5min using the Biorupter™ UCD-200 (Diagenode, Denville, NJ, USA). All experiment was repeated two times.

### Click-IT assays

EU and EdU incorporation was measured using the Click-iT^®^ RNA Alexa Fluor^®^ 488 Imaging and Click-iT^®^ EdU Alexa Fluor^®^ 488 Imaging Kits, respectively (Invitrogen) according to manufacturer protocol. Cells were incubated with EU for 1 hour, 30 or 15 minutes and with EdU for 1 hour before collection for the assay. Experiment was repeated 2-4 times.

### Acidic beta-galactosidase staining

was performed as previously described [52].

### Fluorescent and bright-field microscopy and cell imaging

was done using inverted fluorescent microscope Axio Observer A1 and AxioVision software (Carl Zeiss AG, Oberkochen, Germany).

### Flow cytometry

was performed on LSR Fortessa A and BD LSRII UV A Cytometers (BD Biosciences, San Jose, CA, USA). Obtained data were analyzed by WinList 3D program (Verity Software House, Topsham, ME, USA).

### Microarray hybridization and analyses

Total RNA from cells was isolated using Trizol reagent (Invitrogen). Two biological replicates of each condition were used. RNA processing, labeling and hybridization was done in RPCI Genomics facility using HumanHT-12 v2 Expression BeadChip Kit. Data was analyzed using GeneSpring GX software (Agilent Genomics, Santa Clara, CA, USA).

### Statistical analyses

Unpaired *t*-test was used for comparison of quantitative data between control and experimental groups. LC50 of CBL0137 in cytotoxicity assays was calculated as already described [21]. The associations between SSRP1 and SPT16 expression, LC50 of CBL0137 and sensitivity to FACT knockdown were assessed using the Spearman correlation coefficient. Analyses were conducted using SAS v9.4 (Cary, NC) and all p-values are two-sided.

Analysis of replicates of microarray hybridization was done using Principle Component Analysis built in GeneSpring GX software (Agilent Genomics, Santa Clara, CA, USA).

## SUPPLEMENTARY MATERIALS DATA, FIGURES AND TABLES




