# Supplementary material for: Level of FACT defines the transcriptional landscape and aggressive phenotype of breast cancer cells
Source: Oncotarget. 2017 Feb 23;8(13):20525–42. doi: 10.18632/oncotarget.15656 (PMC5400524; doi:10.18632/oncotarget.15656)
Supplement: Supplementary file 1 [file oncotarget-08-20525-s001.pdf]

Level of FACT defines the transcriptional landscape and aggressive phenotype of breast cancer cells

Supplementary Material

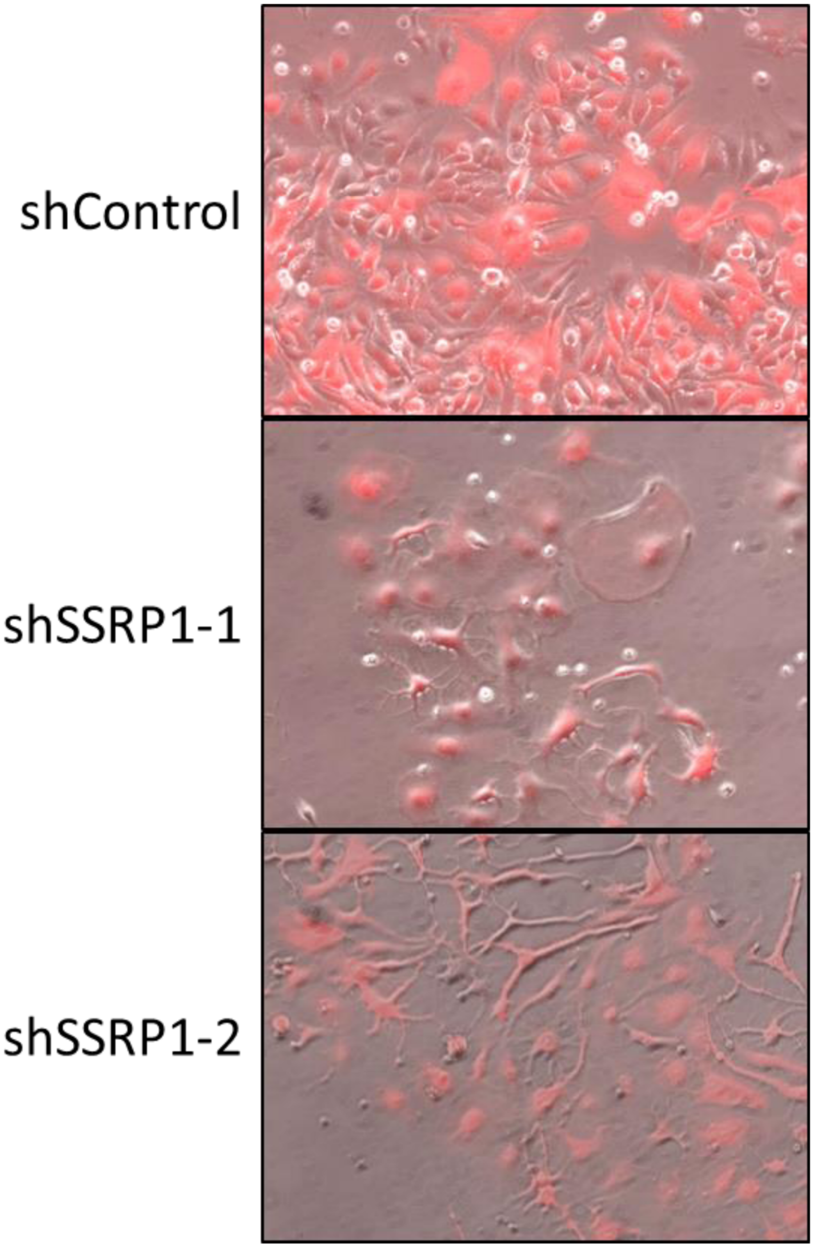

Figure S1. Effect of FACT knockdown on the morphology of MCF7 cell. Cells were transduced with the indicated shRNA bearing lentiviruses also expressing mCherry from an independent promoter. Photographs were taker 8 days after transduction. No selection was done.

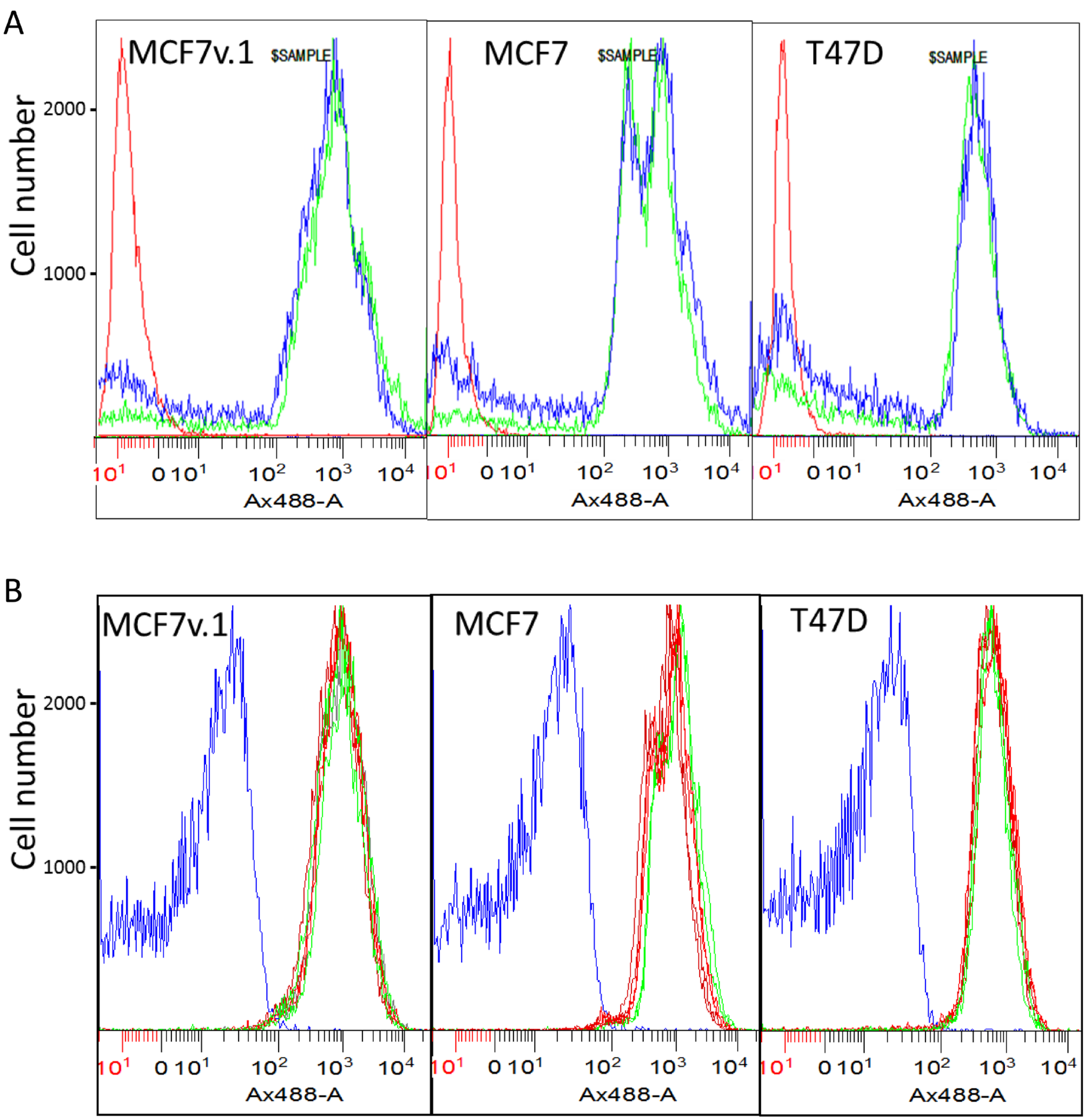

Figure S2. Effect of FACT knockdown on general transcription. A. Flow cytometry analysis of EU incorporation (Ax488-A, 2 hour incubation) into cells transduced with shControl (green), shSSRP1-1 (blue). Mock transduced cells incubated without EU were used as negative control (red) . B. Flow cytometry analysis of EU incorporation (Ax488-A, 15 min incubation) into cells transduced with shControl (green), shSSRP1-1 (red) and mock transduced cells treated with 10ug/ml of Actinomycin D (inhibitor of general transcription) for 4 hours (blue).

A B

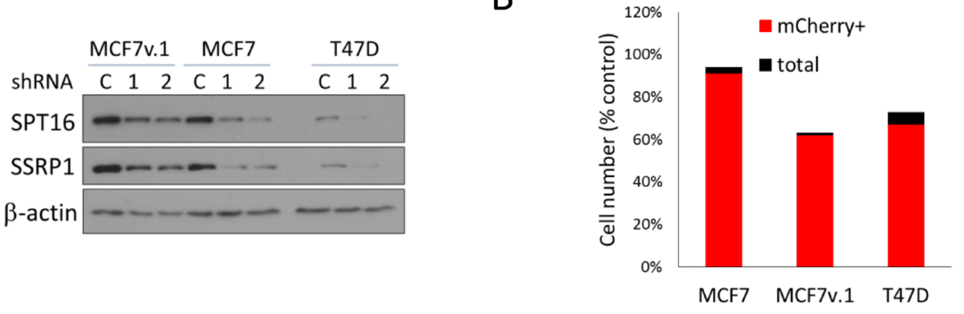

C

Identifier	MCF7-shCo-1	MCF7-shCo-2	MCF7-shSSRP1-1	MCF7-shSSRP1-2	MCF7v.1-shCo-1	MCF7v.1-shCo-2	MCF7v.1-shSSRP1-1	MCF7v.1-shSSRP1-2	T47D-shCo-1	T47D-shCo-2	T47D-shSSRP1-1	T47D-shSSRP-2
MCF7-shCo-1	1.0000	0.9941	0.9767	0.9599	0.9880	0.9899	0.9716	0.9774	0.9762	0.9788	0.9790	0.9540
MCF7-shCo-2	0.9941	1.0000	0.9712	0.9537	0.9896	0.9867	0.9651	0.9710	0.9744	0.9766	0.9789	0.9561
MCF7-shSSRP1-1	0.9767	0.9712	1.0000	0.9790	0.9633	0.9765	0.9725	0.9780	0.9842	0.9855	0.9788	0.9694
MCF7-shSSRP1-2	0.9599	0.9537	0.9790	1.0000	0.9447	0.9598	0.9589	0.9621	0.9680	0.9693	0.9612	0.9562
MCF7v.1-shCo-1	0.9880	0.9896	0.9633	0.9447	1.0000	0.9838	0.9579	0.9643	0.9684	0.9741	0.9786	0.9490
MCF7v.1-shCo-2	0.9899	0.9867	0.9765	0.9598	0.9838	1.0000	0.9746	0.9898	0.9803	0.9826	0.9787	0.9581
MCF7v.1-shSSRP1-1	0.9716	0.9651	0.9725	0.9589	0.9579	0.9746	1.0000	0.9802	0.9620	0.9637	0.9545	0.9411
MCF7v.1-shSSRP1-2	0.9774	0.9710	0.9780	0.9621	0.9643	0.9898	0.9802	1.0000	0.9729	0.9741	0.9624	0.9503
T47D-shCo-1	0.9762	0.9744	0.9842	0.9680	0.9684	0.9803	0.9620	0.9729	1.0000	0.9920	0.9895	0.9820
T47D-shCo-2	0.9788	0.9766	0.9855	0.9693	0.9741	0.9826	0.9637	0.9741	0.9920	1.0000	0.9922	0.9817
T47D-shSSRP1-1	0.9790	0.9789	0.9788	0.9612	0.9786	0.9787	0.9545	0.9624	0.9895	0.9922	1.0000	0.9791
T47D-shSSRP-2	0.9540	0.9561	0.9694	0.9562	0.9490	0.9581	0.9411	0.9503	0.9820	0.9817	0.9791	1.0000

D

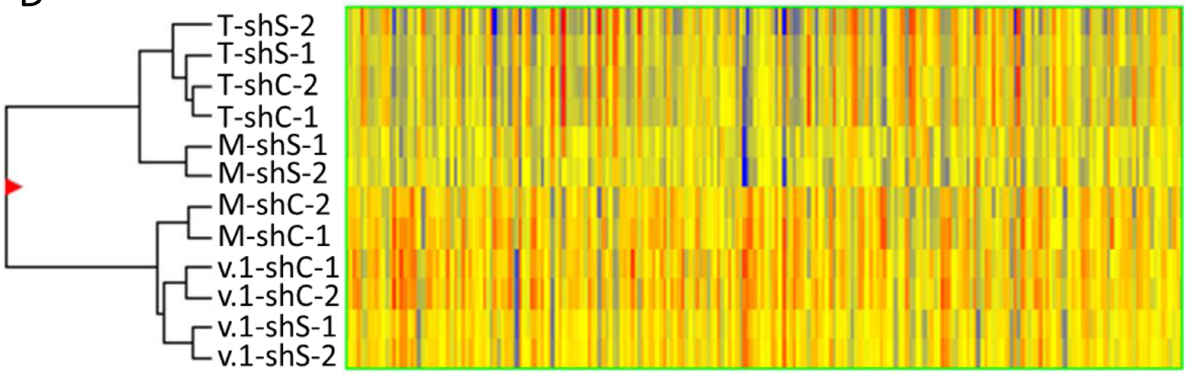

Figure S3. Effect of FACT knockdown on gene expression in BrCa cells. A. Western blotting of extracts of cells prepared in parallel with RNA isolation for microarray hybridization. C – control shRNA, 1 and 2 – shSSRP1-1 and shSSRP1-2. B. Proportion of cells transduced with shRNA to SSRP1 (mCherry+) versus cells transduced with control shRNA 72 hours after transduction. C. Heatmap of correlation coefficients between all samples used for the experiment assessed via principle component analysis. D. Unsupervised hierarchical clustering of all samples and genes whose replicates pass the ANOVA test (p<0.05) using Euclidian distance metric. Dendrogram and heatmap of gene expression with red – high, yellow – median and blue – low level of gene expression across samples.

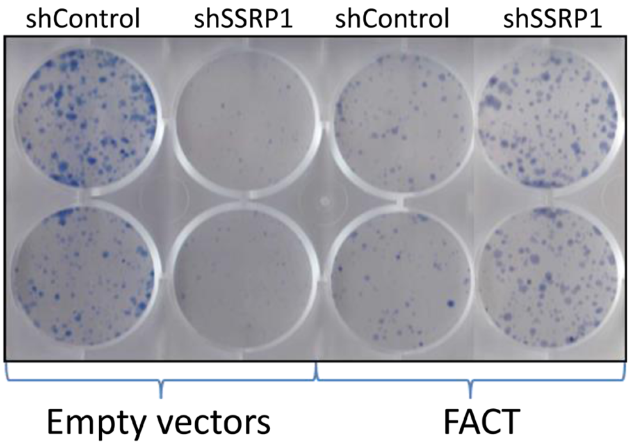

Figure S4. Artificial change of FACT level is toxic for tumor cells. Colonies of Ht1080 cells transduced with either empty vectors or SSRP1 and SPT16 (FACT) in parallel with either control shRNA or shRNA to SSRP1 (shSSRP1-1). ShSSRP1-1 shRNA is targeted against 3'UTR of endogenous SSRP1, while ectopic SSRP1 construct used in this experiment is not sensitive to this shRNA. Note, both reduction of FACT level with shRNA as well as overexpression of SSRP1 lead to the reduced number of colonies, while combination of both neutralizes effect of each other.
